# Blocking amino acid transporter *OsAAP3* improves grain yield by promoting outgrowth buds and increasing tiller number in rice

**DOI:** 10.1111/pbi.12907

**Published:** 2018-03-25

**Authors:** Kai Lu, Bowen Wu, Jie Wang, Wei Zhu, Haipeng Nie, Junjie Qian, Weiting Huang, Zhongming Fang

**Affiliations:** ^1^ Center of Applied Biotechnology Wuhan Institute of Bioengineering Wuhan China; ^2^ National Key Laboratory of Crop Genetic Improvement College of Plant Science and Technology Huazhong Agricultural University Wuhan China

**Keywords:** amino acid, transporter, rice, grain yield, tiller, outgrowth bud

## Abstract

Amino acid transporters (AATs) play indispensable roles in nutrient allocation during plant development. In this study, we demonstrated that inhibiting expression of the rice amino acid transporter *OsAAP3* increased grain yield due to a formation of larger numbers of tillers as a result of increased bud outgrowth. Elevated expression of *OsAAP3* in transgenic plants resulted in significantly higher amino acid concentrations of Lys, Arg, His, Asp, Ala, Gln, Gly, Thr and Tyr, and inhibited bud outgrowth and rice tillering. However, RNAi of *OsAAP3* decreased significantly Arg, Lys, Asp and Thr concentrations to a small extent, and thus promoted bud outgrowth, increased significantly tiller numbers and effective panicle numbers per plant, and further enhanced significantly grain yield and nitrogen use efficiency (NUE). The promoter sequences of *OsAAP3* showed some divergence between *Japonica* and *Indica* rice, and expression of the gene was higher in *Japonica*, which produced fewer tillers than *Indica*. We generated knockout lines of *OsAAP3* on *Japonica* ZH11 and KY131 using CRISPR technology and found that grain yield could be increased significantly. These results suggest that manipulation of *OsAAP3* expression could be used to increase grain yield in rice.

## Introduction

Inorganic nitrogen (N) is mainly absorbed by plants in the form of nitrate and ammonium and is then converted into amino acids directly in the roots or after translocation to the leaves. The amino acids are then transported to roots, leaves, flowers, pollen and embryos (Fischer *et al*., 1998). Amino acids require transporter proteins to move them from source to sink organs (Coruzzi and Bush, 2001; Tegeder, 2012); these amino acid transporters (AATs) are cellular membrane proteins that transport particular amino acids. The transporters play critical roles in various processes in plants such as seed development, and abiotic and pathogen stresses (Näsholm *et al*., 2009; Paungfoo‐Lonhienne *et al*., 2008; Schulze *et al*., 1999).

To date, a large number of *AAT* gene family members have been identified in *Arabidopsis* (Tegeder, 2012), rice (Lu *et al*., 2012), poplar (Wu *et al*., 2015), *Solanum tuberosum* L. (Ma *et al*., 2016) and *Glycine max* L. (Cheng *et al*., 2016). These studies have shown that AAP transporters play important roles in the loading of amino acids for nitrogen sink and supply (Tegeder and Ward, 2012). In *Arabidopsis*, AtAAP1 regulates amino acid transport to root cells and embryos (Lee *et al*., 2007; Sanders *et al*., 2009). In addition, AtAAP1 participates in the uptake of glutamate and neutral amino acids in *Arabidopsis* when these are present at soil concentrations (Perchlik *et al*., 2014). AtAAP2 is localized in the phloem and plays a major role in N transfer from the xylem to phloem (Zhang *et al*., 2010). *AtAAP3* is preferentially expressed in the root phloem (Okumoto *et al*., 2004). AtAAP5 transports amino acids at low concentrations in the roots (Svennerstam *et al*., 2008), and AtAAP6 regulates the amino acid composition of the phloem (Hunt *et al*., 2010). AtAAP8 transports amino acids to the endosperm during early embryogenesis (Schmidt *et al*., 2007) and was recently shown to be localized to the plasma membrane and to function in phloem loading (Santiago and Tegeder, 2016).

Although the functions of AtAATs have been extensively studied in *Arabidopsis*, the roles of OsAATs in rice are much less well understood (Zhao *et al*., 2012). Whole genome analyses have suggested the presence of 79–85 AAT homologous genes in rice (Lu *et al*., 2012; Zhao *et al*., 2012). It has been shown that biomass and yield of rice are altered significantly when *OsAAT* genes are knocked out (Lu *et al*., 2012; Peng *et al*., 2014). At present, it is known that OsAAP6 regulates grain protein content and nutritional quality in rice (Peng *et al*., 2014).

OsAAP3 has activity of transporting Ser, Met, Lys, Leu, His, Gln, Arg, Ala and Gly, especially for transporting the basic amino acids Lys and Arg (Taylor *et al*., 2015). Here, we further indicated that the SNPs in the promoter sequence of *OsAAP3* are divergent between *Japonica* and *Indica* in all 524 rice accession varieties. And the expression level of *OsAAP3* is negatively correlated with tiller number in rice. Blocking *OsAAP3* could improve grain yield by promoting outgrowth buds and increasing tiller numbers especially in rice *Japonica* through regulating the concentrations of Lys, Arg, His, Asp, Ala, Gln, Gly, Thr and Tyr.

## Results

### Sequence divergence in the *OsAAP3* promoter regions of *Japonica* and *Indica* rice

**Figure 1:**
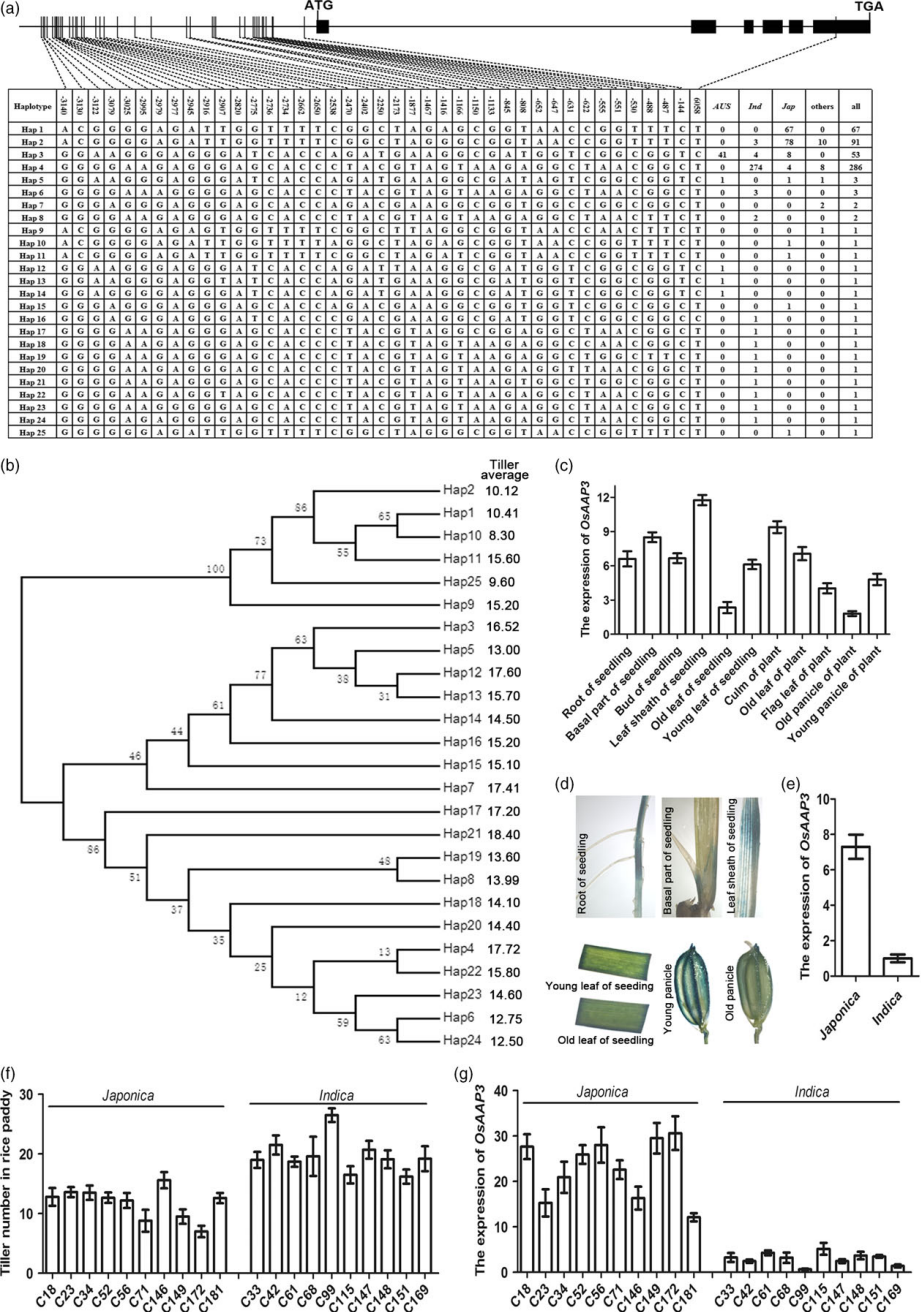
The promoter sequence of *OsAAP3* has SNP divergence between rice *Japonica* and *Indica*, and its expression level is associated with rice tiller. (a) SNP divergence in the promoter sequence of *OsAAP3*. (b) The phylogeny of promoter sequence of *OsAAP3*. (c) The expression level of *OsAAP3* in different tissues in ZH11. (d) The root, basal part, leaf sheath, young leaf, old leaf, young panicle and old panicle GUS staining using p*O* *sAAP3::GUS* transgenic plant. (e) The average expression level of *OsAAP3* in the culm of rice *Japonica* and *Indica* in a diverse worldwide collection of 524 *O. sativa* landraces (Chen *et al*., 2014). (f) Tiller number in ten cultivars *Japonica* and ten cultivars *Indica*. (g) The expression of *OsAAP3* in the culm of ten cultivars *Japonica* and ten cultivars *Indica*.

Overall, 524 rice accession varieties were used in this study, and these belong to nine subpopulations: *IndI*,* IndII*,* Indica* intermediate, *Tej*,* Trj*,* Japonica* intermediate, *Aus*,* VI* and intermediate (Chen *et al*., 2014). The *Indica* subpopulation (*IndI*,* IndII* and *Indica* intermediate) included 295 accessions, while the *Japonica* subpopulation (*Tej*,* Trj* and *Japonica* intermediate) included 156 accessions. We analysed the exons and promoter sequences of *OsAAP3* in all 524 accessions and identified 25 haplotypes in the accessions (Figure 1a). The Hap1 and Hap2 haplotypes were mainly present in *Japonica* accessions; the Hap4 haplotype was mainly found in *Indica* rice (Figure 1a). The Hap1/2 and Hap4 haplotypes belonged to two separate evolutionary branches (Figure 1b). An average tiller number of 11 was found in accessions with the Hap1/2 haplotypes, but was 17.72 in accessions with a Hap4 haplotype (Figure 1b).

*OsAAP3* was mainly expressed in root, leaf, leaf sheath, culm and panicle (Figure 1c), especially in the root elongation area for lateral root growth, the basal part of the culm for bud outgrowth, the young leaf and the young panicle (Figure 1d). *OsAAP3* showed higher expression level in the culm of *Japonica* accessions that carried the Hap1/2 haplotypes compared with *Indica* accessions with the Hap4 haplotype (Figure 1e). We also found that the expression level of *OsAAP3* was negatively correlated with tiller number (Figure 1f–g). The *Japonica* accessions had lower tiller numbers (Figure 1f), which was associated with the higher level of *OsAAP3* expression in the culm (Figure 1g).

### Down‐regulation of *OsAAP3* expression boosts grain yield by increasing tiller numbers in rice

**Figure 2:**
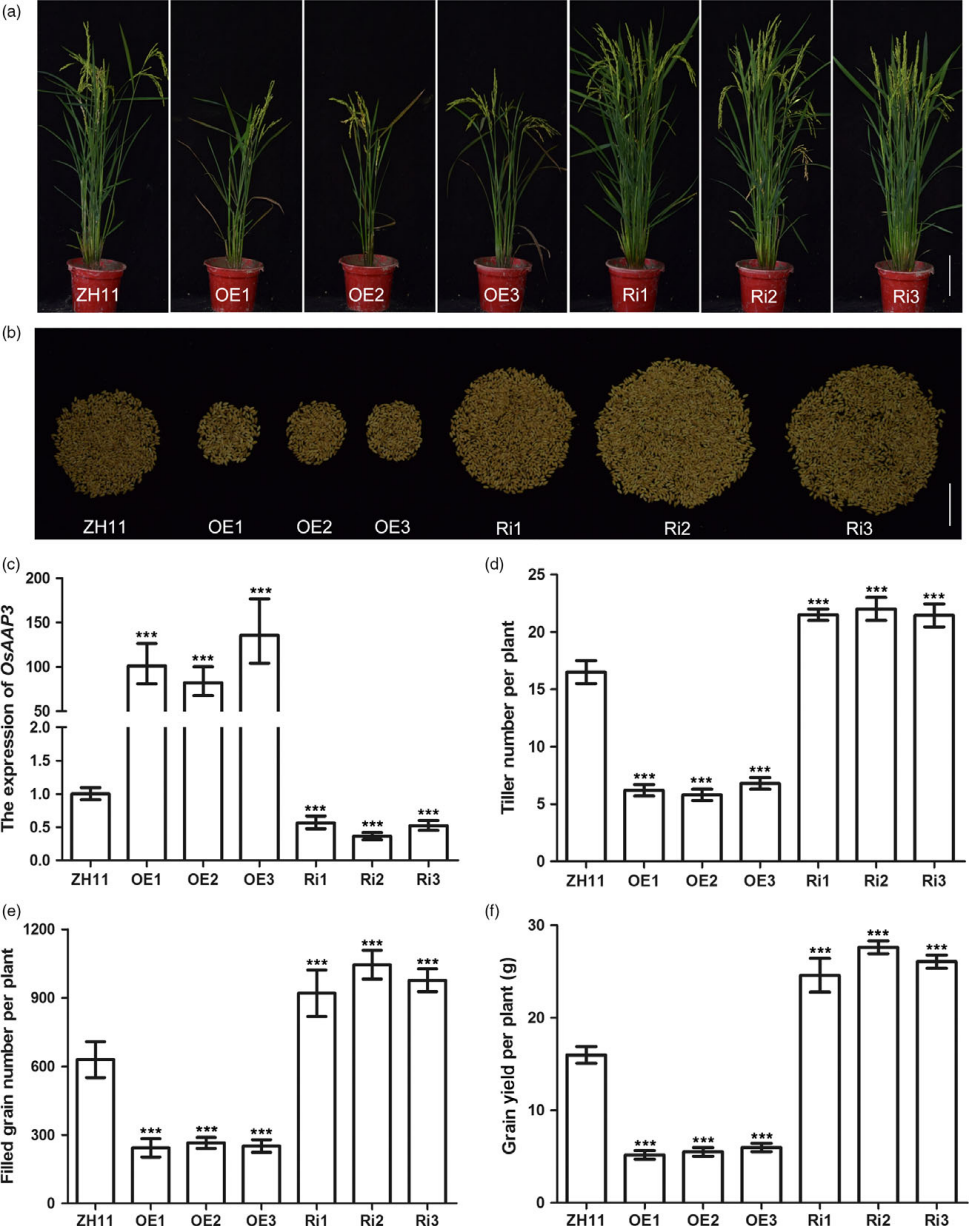
Phenotypes of the rice plants with altered expression of *OsAAP3* grown in paddy field. Whole plant phenotype (a) and filled grain number per plant (b) of wild‐type ZH11, *OsAAP3* overexpressing lines (OE1‐OE3) and *OsAAP3*‐RNAi lines (Ri1‐Ri3). The expression of *OsAAP3* in leaf (c), tiller number per plant (d), filled grain number per plant (e) and grain yield per plant (f) ZH11, OE1‐OE3 lines and Ri1‐Ri3 lines. Scale bars, 15.0 cm in (a) and 5.0 cm in (b). Values are means ± SD (*n* > 30). Significant levels: ***P < 0.001; **P < 0.01; * *P* < 0.05.

To analyse the function of *OsAAP3* in rice plants, we generated OE (overexpression) and Ri (RNA interference) transgenic lines for *OsAAP3* under the control of rice 35S and *Ubi‐1* promoters, respectively. Tiller numbers in *OsAAP3* OE lines (OE1, OE2 and OE3) were comparatively lower than that in wild‐type ZH11 plants (Figure 2a, d). However, tiller numbers in *OsAAP3* Ri lines (Ri1, Ri2 and Ri3) were significantly higher than that in ZH11 plants (Figure 2a, d). RT‐PCR analysis of leaves revealed that three OE lines had relatively high levels of *OsAAP3* transcripts, whereas three Ri lines had low levels of *OsAAP3* transcripts (Figure 2c). To determine the effects of altered expression of *OsAAP3* on agronomic traits related to grain yield, we measured filled grain numbers per plant and grain yield per plant of OE and Ri lines in the field (Figure 2e, f). We found that filled grain numbers per plant (Figure 2e) and grain yield per plant (Figure 2f) in OE lines fell to less than half of that in ZH11, but increased significantly in Ri lines compared to that in ZH11 (Figure 2e, f).

### Down‐regulation of *OsAAP3* expression promotes bud elongation

**Figure 3:**
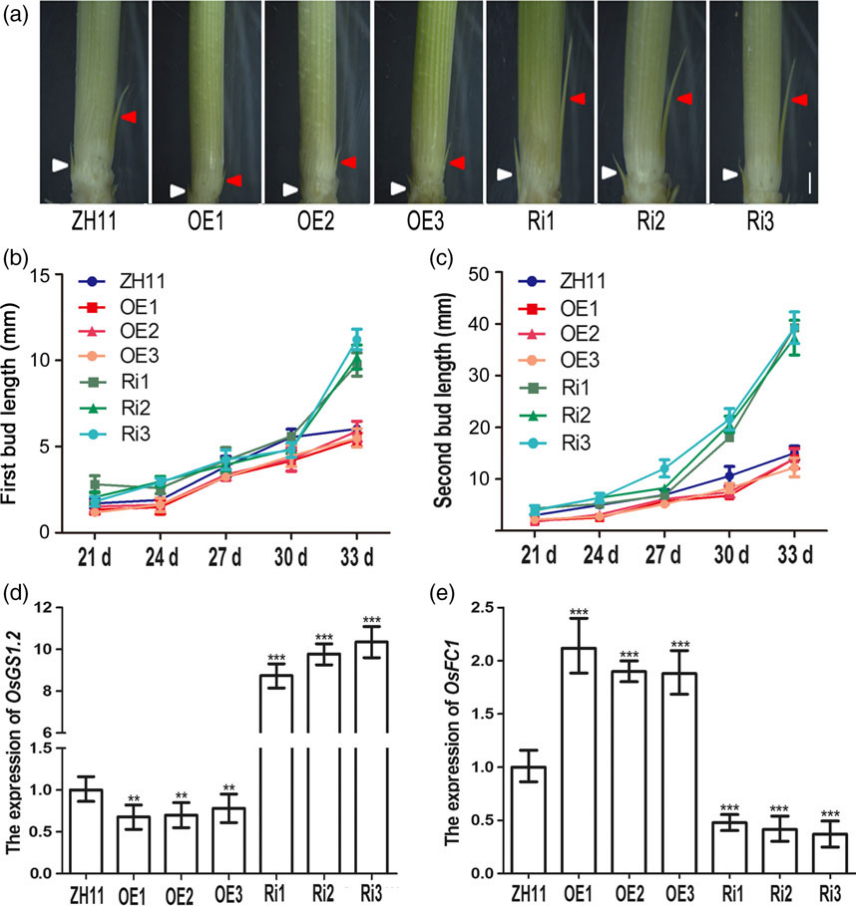
Phenotypes of outgrowth bud with altered expression of *OsAAP3* grown in hydroponic culture. (a) Phenotypes of outgrowth bud of ZH11, OE1‐OE3 lines and Ri1‐Ri3 lines. Kinetic analyses of the elongation of first outgrowth bud (b) and second outgrowth (c) at the seedlings basal part of ZH11, OE1‐OE3 lines and Ri1‐Ri3 lines. The expression of bud elongation‐related genes *OsGS1.2* (d) and *OsFC1* (e) in the axillary buds of ZH11, OE1‐OE3 lines and Ri1‐Ri3 lines. Scale bars, 1.0 mm in (a). Values are means ± *SD* (*n* > 20). Significant levels: ***P < 0.001; **P < 0.01; *P < 0.05.

In rice, the tiller is produced by bud outgrowth at the vegetative stage and involves two developmental stages: bud formation and bud elongation (Arite *et al*., 2009). To determine how *OsAAP3* affected tiller development, we measured bud formation and elongation in *OsAAP3* OE and Ri lines. Plants grown under hydroponic conditions with 2.0 mm ammonium nitrate produced buds. However, bud elongation was significantly slower in OE lines than that in ZH11 (Figure 3a), while bud length in Ri lines was significantly greater than that in ZH11 (Figure 3a). We also investigated the dynamic process of bud elongation in OE and Ri lines and found that the first buds in the Ri lines grew more rapidly than that in OE lines or ZH11; there was a clear significant difference between the different genotypes at 33 days after seed germination (DAG, Figure 3b). Similarly, the second buds in Ri lines also grew more rapidly than that in ZH11 and were clearly larger at 30 DAG; by contrast, OE lines were consistently behind that in ZH11 plants (Figure 3c). The genes *OsGS1.2* and *OsFC1* have been shown to be related to axillary bud outgrowth (Minakuchi *et al*., 2010; Ohashi *et al*., 2017). The expression of *OsGS1.2* (Figure 3d) decreased in OE lines and increased in Ri lines at the axillary buds. However, *OsFC1* (Figure 3e) was found to show up‐regulated expression in OE lines and down‐regulated expression in Ri lines at the axillary buds. In addition, root length was decreased significantly under low‐nitrogen conditions (0.5–2.0 mm) in OE lines (Figure S1a‐d), but was increased significantly under 1.0 mm and 8.0 mm nitrogen concentrations in Ri lines (Figure S1a‐d). Root numbers (Figure S1a‐c, e) and plant height (Figure S1a‐c, f) also increased significantly under 1.0–8.0 mm nitrogen in Ri lines. These results indicated that RNAi‐mediated down‐regulation of *OsAAP3* promoted bud outgrowth and seedling growth and might trigger the formation of an increased tiller number.

### Effect of *OsAAP3* expression on amino acid composition in rice straw and grain

**Figure 4:**
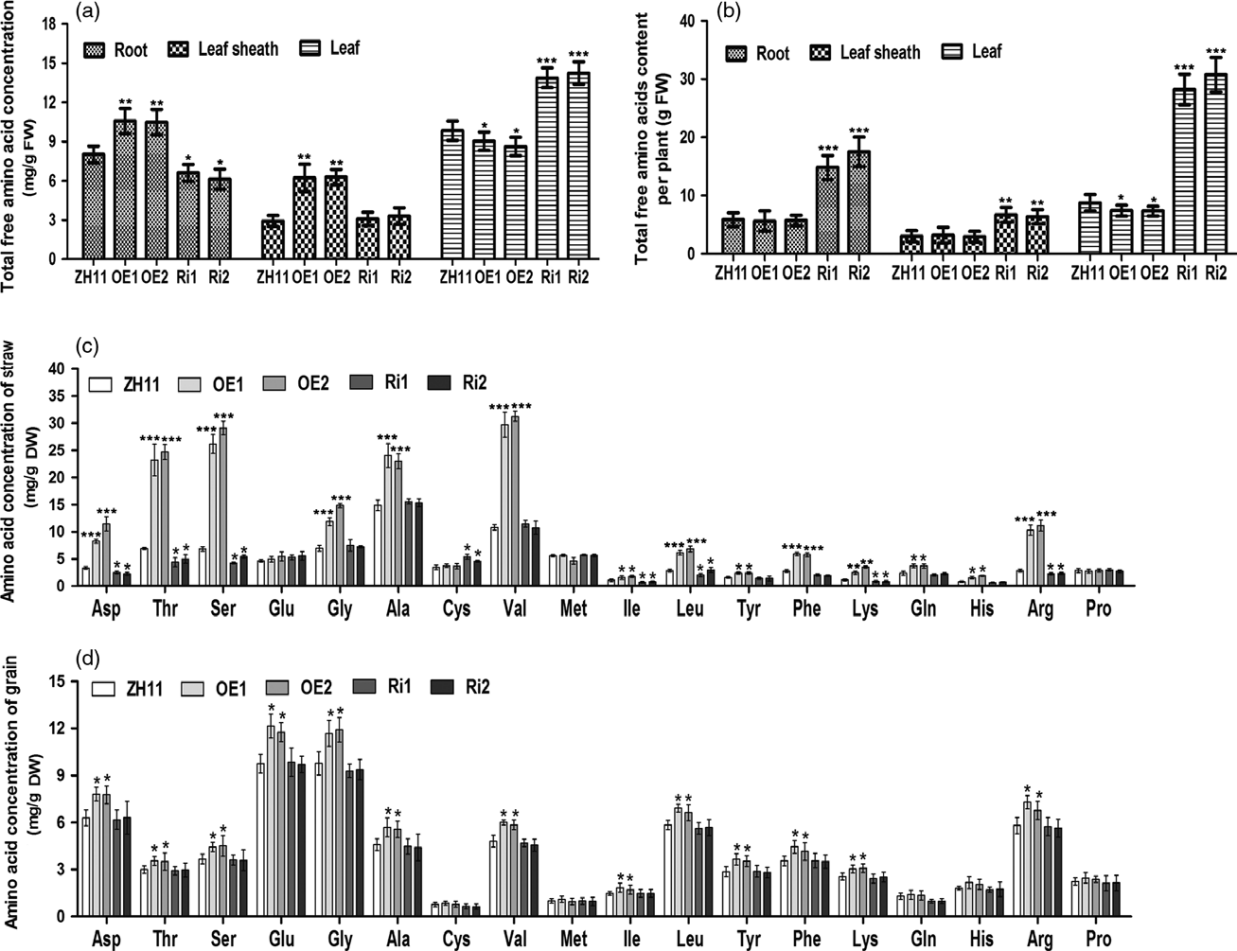
Effect of *OsAAP3* on amino acid content in ZH11, *OsAAP3* overexpressing lines (OE1‐OE2) and *OsAAP3*‐RNAi lines (Ri1‐Ri2). Total free amino acid concentration (a) and total free amino acid content per plant (b) in root, leaf sheath, leaf of ZH11, OE1‐OE2 lines and Ri1‐Ri2 lines at seedling stage. Amino acid concentration of straw at filling stage (c) and grain at mature stage (d) in ZH11, OE1‐OE2 lines and Ri1‐Ri2 lines grown in paddy. Values are means ± SD (*n* > 3). Significant levels: ***P < 0.001; **P < 0.01; *P < 0.05.

As OsAAP3 is an amino acid transporter, we tested the effects of altering *OsAAP3* expression levels on amino acid composition in rice straw and grain using the ninhydrin and HPLC method (Figure 4). In seedlings, total free amino acid concentrations in OE lines were increased significantly in roots and leaf sheaths, but decreased significantly in leaves (Figure 4a). By contrast, total free amino acid concentrations in Ri lines were decreased significantly in roots and leaf sheaths but increased significantly in leaves (Figure 4a). In addition, the total amount of amino acids in each tissue of OE line plants did not exceed that of ZH11 plants (Figure 4b), although the amino acid content in each tissue of Ri line plants was much higher than that in ZH11 plants (Figure 4b). To determine why amino acid concentrations were higher in OE lines, but overall amino acid content was lower, we measured the levels of individual amino acids in straw from OE and Ri lines. We found that the concentrations of Asp, Thr, Ser, Gly, Ala, Val, Ile, Leu, Tyr, Phe, Lys, Gln, His and Arg were increased significantly in OE lines (Figure 4c). However, the concentrations of Asp, Thr, Ser, Ile, Leu, Lys and Arg were decreased significantly in Ri lines (Figure 4c). A similar analysis of grains showed that the concentrations of Asp, Thr, Ser, Glu, Gly, Ala, Val, Ile, Leu, Tyr, Phe, Lys and Arg were increased significantly in OE lines (Figure 4d). However, none of the measured amino acids decreased in concentrations in grains of Ri lines (Figure 4d). These results showed that overexpression of *OsAAP3* could increase the concentrations of amino acids, which inhibited the growth of plants; however, a reduction in expression of *OsAAP3* prevented any inhibition of growth but did not affect the nutritional quality of the grain.

### Increased concentrations of partial amino acids inhibit bud elongation and rice growth

**Figure 5:**
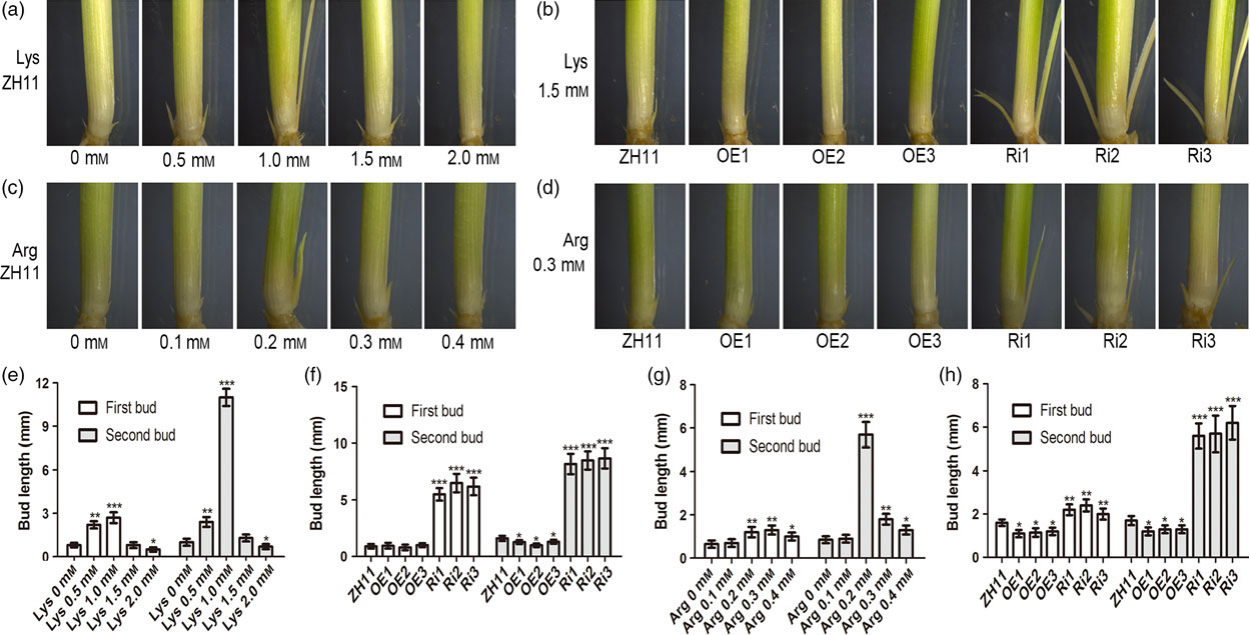
Effect of amino acid Lys and Arg on outgrowth bud elongation of the rice seedlings grown in hydroponic culture. Phenotypes (a) and bud length (e) of outgrowth bud in ZH11 under 1.0 mm NH_4_ NO_3_ containing different Lys concentrations (0 mm, 0.5 mm, 1.0 mm, 1.5 mm, 2.0 mm). Phenotypes (b) and bud length (f) of outgrowth bud in ZH11, OE1‐OE3 lines, and Ri1‐Ri3 lines under 1 mm NH_4_ NO_3_ containing 1.5 mm Lys. Phenotypes (c) and bud length (g) of outgrowth bud in ZH11 under 1.0 mm NH_4_ NO_3_ containing different Arg concentrations (0 mm, 0.1 mm, 0.2 mm, 0.3 mm, 0.4 mm). Phenotypes (d) and bud length (h) of outgrowth bud in ZH11, OE1‐OE3 lines and Ri1‐Ri3 lines under 1.0 mm NH_4_ NO_3_ containing 0.3 mm Arg. The proportion of (a)‐(d) is the same size. Values are means ± SD (*n* > 15). Significant levels: ***P < 0.001; **P < 0.01; * *P* < 0.05.

As shown above, elevated expression of *OsAAP3* caused an accumulation of amino acids, especially of Lys and Arg (Figure 4c), which can be directly transported by OsAAP3 (Taylor *et al*., 2015). However, bud elongation and plant growth were reduced in plants with elevated amino acids levels. Interestingly, our results showed that expression of *OsAAP3* could be induced by exogenous Lys and Arg as its expression level peaked in roots after an 8‐h treatment (Figure S2a) and after 12 h in the basal part of the leaf sheath (Figure S2b). To understand the effects of these amino acids on plant growth and development, we added different amino acids to the nutrient solution in which wild‐type ZH11 plants were growing. We found that low concentrations of Lys (0–1.0 mm) could promote the elongation of buds in rice (Figure 5a, e), but high concentrations (1.0–2.0 mm) inhibited elongation of buds, especially for second buds (Figure 5a, e). In the transgenic lines, bud elongation was inhibited in OE lines (Figure 5b, f), but was accelerated in Ri lines (Figure 5b, f), suggesting that the inhibitory effect was removed. In a similar fashion, high concentrations of Arg (0.2–0.4 mm) inhibited bud elongation (Figure 5c, g), whereas low concentrations (0–0.2 mm) increased bud elongation (Figure 5c, g). The inhibitory effect on bud growth could be prevented in Ri lines by treatment with 0.3 mm Arg (Figure 5d, h), although the effect was not as strong as seen for Lys treatment. We also indicated that very low concentrations (0–0.5 mm) of Asp, Ser, Gly and Tyr (Figure S3), low concentrations (0–1.0 mm) of Thr, Ala, Val, Leu and Gln (Figure S4) and medium concentrations (0–2.0 mm) of Ile Phe and His (Figure S5) could promote the elongation of buds in rice, but enhanced concentrations of those amino acids inhibited the elongation of buds, especially for second buds (Figures S3‐S5). In the transgenic lines, bud elongation was inhibited in OE lines, but was accelerated in Ri lines with the treatments of Asp, Gly, Tyr (Figure S3), Thr, Ala, Gln (Figure S4) and His (Figure S5). Bud elongation of all transgenic lines was accelerated under the treatments of Ile, Phe and Leu, but was inhibited under the treatments of Ser and Val, suggesting that the transgenic plants of *OsAAP3* have no physiological effects under amino acids Ile, Phe, Leu, Ser and Val.

Root length, root number and plant height were decreased significantly by a 1.5 mm Lys treatment in OE lines (Figure S6a, c‐e); however, all three traits increased in similarly treated Ri lines, especially root number and plant height (Figure S6a, c‐e). Root length, root number and plant height were decreased significantly by a 0.3 mm Arg treatment in OE lines (Figure S6b, f‐h), but were increased significantly in Ri lines (Figure S6b, f‐h). The results described above indicate that Lys and Arg are not only amino acids absorbed by plants but also play important roles in regulating the growth and development of plants. Shoot branching (tiller) and growth are regulated by plant hormones, particularly cytokinins (CKs) (Ferguson and Beveridge, 2009). Levels of cytokinins were regulated through the irreversible oxidative cleavage of the N^6^‐side chain by CYTOKININ DEHYDROGENASE/OXIDASE (CKXs) (Zurcher and Muller, 2016). To investigate the possible interaction with CKs, we measured the gene expression of 11 *CKXs* and found most of *OsCKXs* (*OsCKX2*,* OsCKX3*,* OsCKX4*,* OsCKX5*,* OsCKX6*,* OsCKX8*,* OsCKX9* and *OsCKX10*) exhibited higher expression level in OE lines compared to that in ZH11, whereas most of *OsCKXs* (*OsCKX2*,* OsCKX3*,* OsCKX5*,* OsCKX6*,* OsCKX8*,* OsCKX9*,* OsCKX10* and *OsCKX11*) demonstrated lower expression level in both Ri lines and mutant than that in ZH11 (Figure S7). These results suggest that altered expression of *OsAAP3* controlled axillary bud outgrowth possibly by regulating CK pathway in the axillary bud.

### Down‐regulation of *OsAAP3* expression enhances nitrogen use efficiency

**Figure 6:**
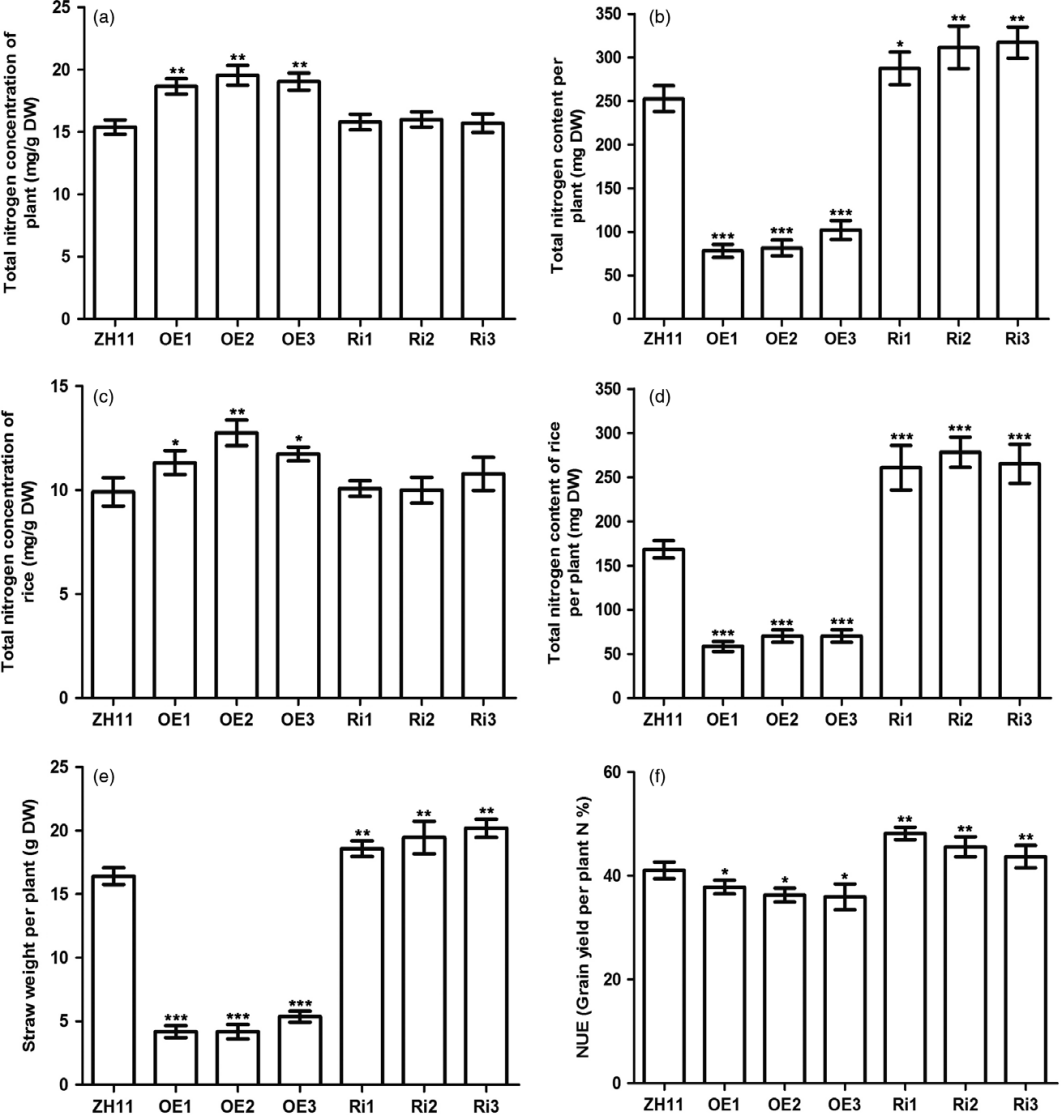
Effect of *OsAAP3* on nitrogen content in ZH11, *OsAAP3* overexpressing lines (OE1‐OE3) and *OsAAP3*‐RNAi lines (Ri1‐Ri3). (a) Total nitrogen concentrations of straw of ZH11, OE1‐OE3 and Ri1‐Ri3 line at filling stage grown in paddy. (b) Total nitrogen content per plant of ZH11, OE1‐OE3 and Ri1‐Ri3 lines at filling stage grown in paddy. (c) Total nitrogen concentrations of grain of ZH11, OE1‐OE3 and Ri1‐Ri3 lines at filling stage grown in paddy. (d) Total nitrogen content of grain of ZH11, OE1‐OE3 and Ri1‐Ri3 line at filling stage grown in paddy. (e) Straw dry weight of ZH11, OE1‐OE3 and Ri1‐Ri3 lines at mature stage grown in paddy. (f) Nitrogen use efficiency (NUE) of ZH11, OE1‐OE3 and Ri1‐Ri3 lines at mature stage grown in paddy. Values are means ± SD (*n* > 3). Significant levels: ***P < 0.001; **P < 0.01; * *P* < 0.05.

To investigate the role of *OsAAP3* in nitrogen use efficiency (NUE), total N concentration was measured. Overexpression of *OsAAP3* led to a higher total N concentration, whereas total N concentration was unchanged after down‐regulation of *OsAAP3* expression (Figure 6a). Total N content per plant in OE lines was lower than that in ZH11, but higher than in Ri lines (Figure 6b). To confirm that *OsAAP3* participates in N transportation to the panicle, the total N levels of mature seeds in the transgenic rice lines were measured (Figure 6c, d). Total N concentration in mature seeds was increased in OE lines, but no significant difference was found between Ri lines and wild‐type ZH11 (Figure 6c). Thus, total N content of rice seeds in OE lines was decreased, but increased significantly in Ri lines (Figure 6d). The straw dry weight per plant of OE lines was significantly lower than that of ZH11 plants (Figure 6e). By contrast, down‐regulation of *OsAPP3* expression increased significantly straw dry weight per plant (Figure 6e). The NUE of OE lines was decreased significantly relative to ZH11 plants, but was increased significantly in Ri lines (Figure 6f).

### Down‐regulation of *OsAAP3* by CRISPR technology increases grain yield in both *Japonica* ZH11 and KY131

**Figure 7:**
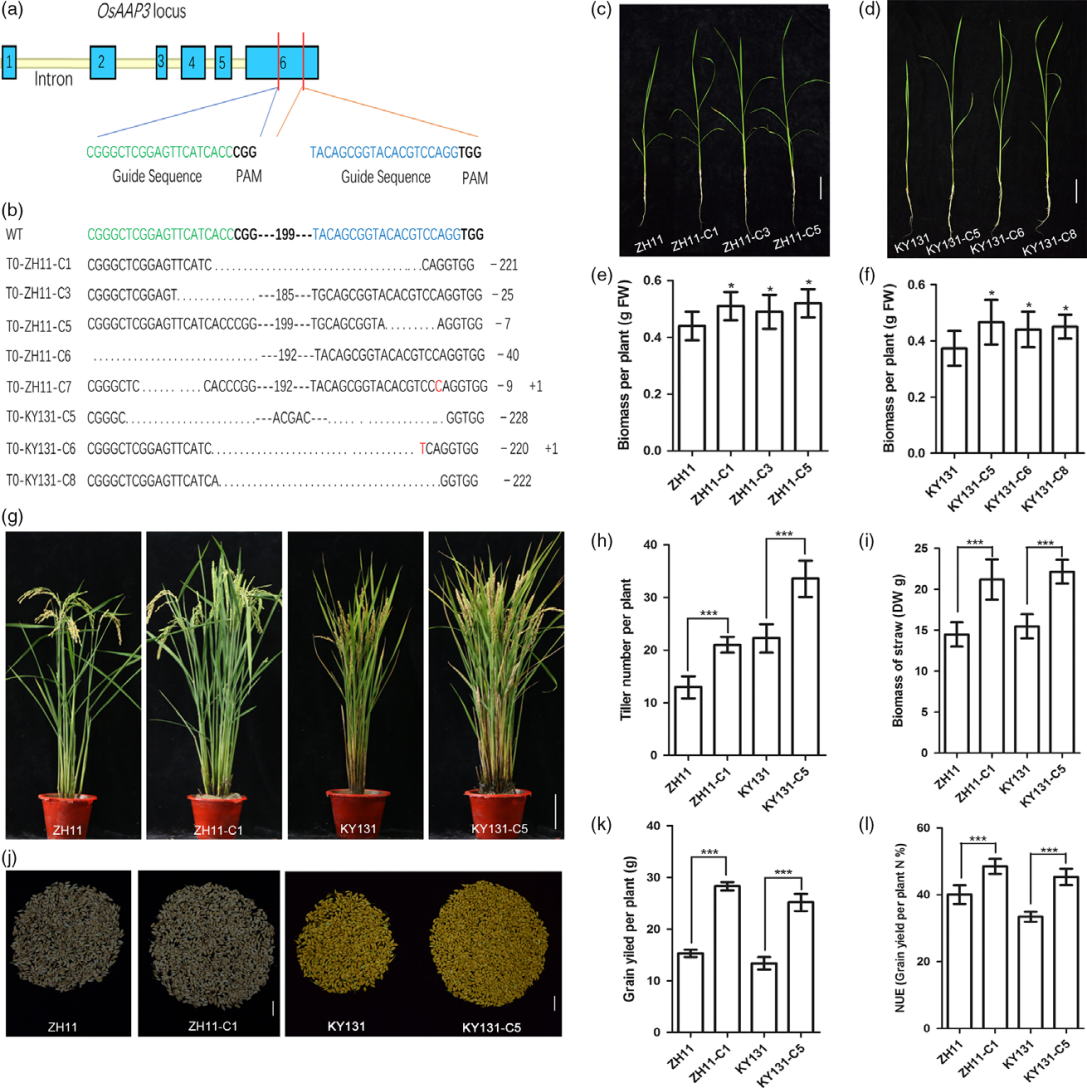
Blocking *OsAAP3* improves grain yield by increasing tiller number in rice. (a) Target 1 and target 2 of gene knockout for *OsAAP3* with CRISPR technology. (b) Sequencing results of base deletion and insertion of *OsAAP3*‐CRISPR in *Japonica* ZH11 and KY131. On the right, minus (–) and plus (+) signs indicate the number of nucleotides deleted and inserted at *OsAAP3*‐CRISPR target sequence site 1 and site 2, respectively. Phenotypes of *OsAAP3* CRISPR lines (three) in ZH11 (c) and in KY131 (d). Biomass analyses of *OsAAP3* CRISPR lines (three) in ZH11 (e) and in KY131 (f). Whole plant phenotype of *OsAAP3* CRISPR line in ZH11 and in KY131 (g). Tiller number per plant (h), biomass of straw (i), grain yield per plant (j‐k) and NUE (l) analyses of *OsAAP3*‐CRISPR line in ZH11 and in KY131. Scale bars, 3 cm in (c), 3 cm in (d), 15 cm in (g) and 2.5 cm in (j). Values are means ± SD (*n* > 20). Significant levels: ***P < 0.001; **P < 0.01; * *P* < 0.05.

Kongyu 131 (KY131) is a *Japonica* variety with the largest planting area in China. We compared the expression level of *OsAAP3* in *Japonica* ZH11 and KY131 and found a higher expression level in KY131 (Figure S8). We knocked out the *OsAAP3* sequence in *Japonica* ZH11 and KY131 using CRISPR technology, as higher expression level of *OsAAP3* limited rice tiller numbers. Targets 1 and 2 for knockout of the *OsAAP3* gene with CRISPR technology are shown in Figure 7a, and sequencing results showing base deletions and insertions after *OsAAP3*‐CRISPR in *Japonica* ZH11 and KY131 are shown in Figure 7b. The effects of *OsAAP3* knockout on seedling growth and agronomic traits related to grain yield were measured in *OsAAP3*‐CRISPR lines. The biomasses of *OsAAP3*‐CRISPR lines ZH11‐C and KY131‐C were greater than those of ZH11 or KY131 (Figure 7c–f). Tiller number (Figure 7g–h), biomass of straw (Figure 7i), grain yield (Figure 7j–k) and NUE (Figure 7l) were also increased significantly in *OsAAP3*‐CRISPR lines both in ZH11 or KY131 background compared with each wild‐type.

**Figure 8:**
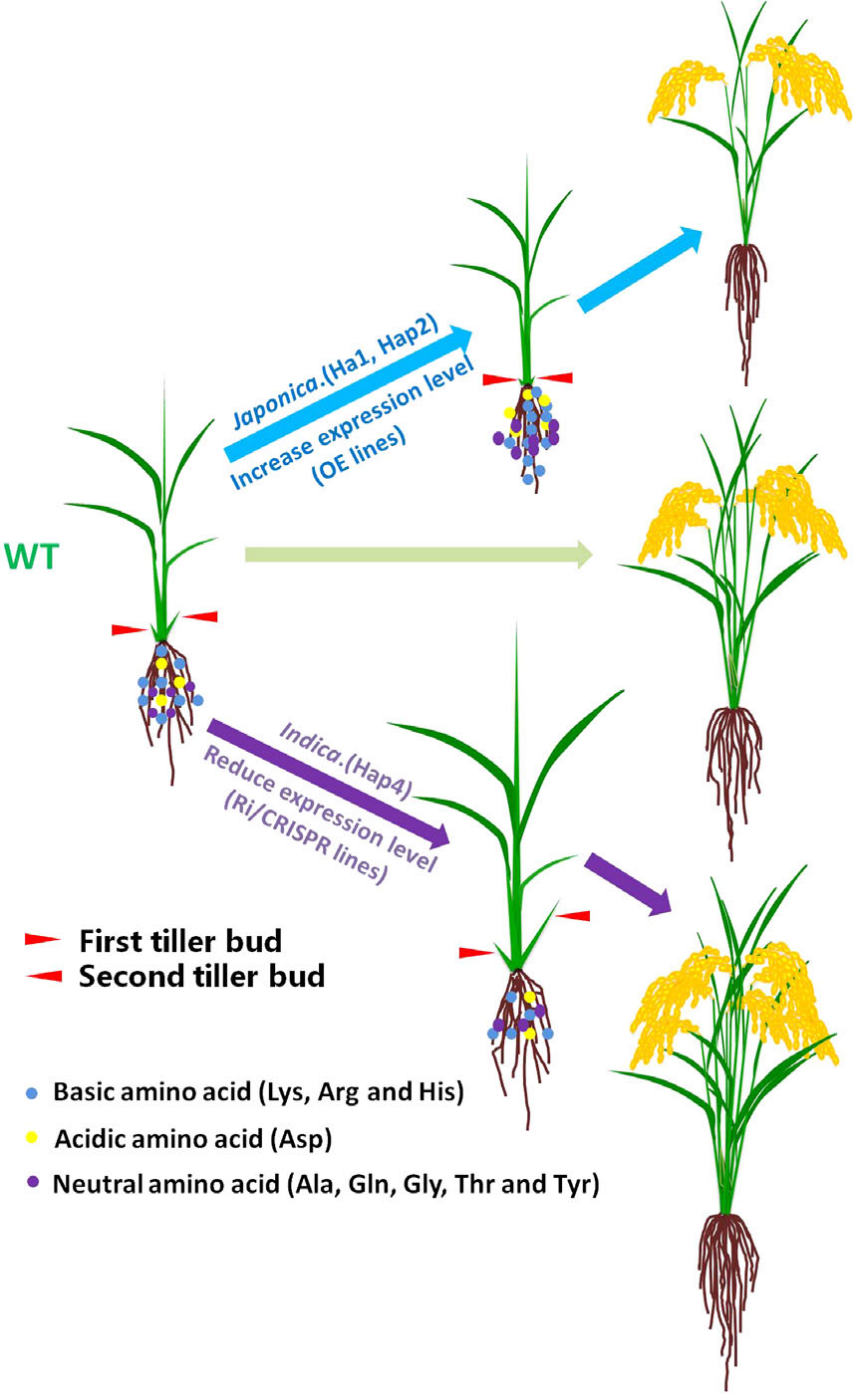
The proposed model of *OsAAP3* regulating rice tillering and grain yield. In OE or *Japonica* lines, elevated expression of *OsAAP3* accumulates basic amino acid (Lys, Arg and His), acidic amino acid (Asp), neutral amino acid (Ala, Gln, Gly, Thr and Tyr). Hence, axillary bud is inhibited, which is unfavourable for rice tillering. However, RNAi/CRISPR line or *Indica* of *OsAAP3* induces the opposite effects. The red arrows mean first tiller bud and second tiller bud, the blue arrows mean the development of rice OE lines or *Japonica* (such as KY131) from seedling to mature plant, the green arrow means the development of rice wild type (such as *Japonica* ZH11) from seedling to mature plant, and the pink arrows mean the development of rice RNAi/CRISPR line or *Indica* from seedling to mature plant. The blue circles mean basic amino acid (Lys, Arg and His), the yellow circles mean acidic amino acid (Asp), and the pink circles mean neutral amino acid (Ala, Gln, Gly, Thr and Tyr).

Based on these results, we propose a model in which *OsAAP3* participates in elongating outgrowth bud and increasing rice tiller number to modify grain yield through regulation the concentrations of Lys, Arg, His, Asp, Ala, Gln, Gly, Thr and Tyr in rice (Figure 8). In OE lines or *Japonica*, elevated expression of *OsAAP3* may accumulate the concentrations of these amino acids. Hence, axillary bud is inhibited, which is unfavourable for rice tillering. However, *Indica* or RNAi/CRISPR line of *OsAAP3* induces the opposite effects.

## Discussion

### Reduced expression of *OsAAP3* can increase significantly tiller numbers, but does not change the nutritional quality of rice grains

Rice tillering is an important agronomic trait as it determines panicle number and grain yield (Li *et al*., 2003a,b; Xing and Zhang, 2010). In our study, we found that tiller numbers increased significantly in Ri lines at the productive stage (Figure 2a, b, d) compared with wild‐type ZH11 and were also elevated in *OsAAP3‐*CRISPR lines compared with wild‐type ZH11 or KY131 (Figure 7g, h). By contrast, tiller numbers were reduced significantly in OE lines (Figure 2a, b, d). Compared to wild‐type, Ri lines had a higher filled grain number per plant (Figure 2b, e), whereas OE lines had a reduced filled grain numbers per plant (Figure 2b, e). Our results also indicated that reduced expression of *OsAAP3* could enhance significantly grain yield per plant (Figure 2f). *OsAAP3‐*CRISPR lines gave similar results as described above and showed an increased grain yield per plant (Figure 7j, k). Similarly, *Arabidopsis ataap2* mutants increase branch and silique numbers per plant and seed yield are strongly increased (Zhang *et al*., 2010).

Rice nutritional quality is another important trait that is used in breeding of new rice varieties (Zhang, 2007). Rice grain quality includes eating and cooking quality, nutritional quality, milling quality and appearance quality (Li *et al*., 2003a,b). The rice amino acid transporter OsAAP6 regulates starch storage and also affects protein storage in rice endosperm (Peng *et al*., 2014). In our study, a reduction in the level of expression of *OsAAP3* did not cause any change in amino acid concentrations in grains (Figure 4d). Additionally, total nitrogen concentration was unchanged compared with wild‐type ZH11 (Figure 6d). Plant membrane transporter genes can be incorporated into programs to enhance crop yields (Schroeder *et al*., 2013). Therefore, our systematic study of the function of transporter OsAAP3 is important for future genetic improvements in rice to increase grain yield and nutritional quality.

### Partial amino acid concentrations influence bud outgrowth and tiller number in rice through the amino acid transporter OsAAP3

Rice tillers are produced by shoot branching, which consists of two distinct steps: first, formation of an axillary bud at each leaf axil; and second, outgrowth of the axillary bud (Li *et al*., 2003a,b; Xing and Zhang, 2010). Therefore, final tiller number is determined not only by how many tiller buds are formed but also by how many tiller buds are capable of outgrowth (Wang and Li, 2011). Axillary bud outgrowth is regulated by environmental signals (Xing and Zhang, 2010). Nitrogen is a crucial determinant of plant growth and crop productivity (Hachiya and Sakakibara, 2017; Li *et al*., 2017). Plants make use of transporters to take up N from the soil via the roots and transport it to other organs. Our study indicated that low concentrations of amino acids could promote the elongation of axillary buds in rice (Figure 5; Figures S3‐S5), and that high concentrations of amino acids could inhibit axillary bud elongation, especially for second buds (Figure 5; Figures S3‐S5). OsAAP3 has activity of transporting Ser, Met, Lys, Leu, His, Gln, Arg, Ala and Gly, especially for transporting the basic amino acids Lys and Arg (Taylor *et al*., 2015). In the present study, we found that elongation of buds was inhibited by higher concentrations of Lys, Arg, His, Asp, Ala, Gln, Gly, Thr and Tyr in both OE lines and ZH11 (Figure 5; Figures S3‐S5), but was increased in Ri lines (Figure 5; Figures S3‐S5). These results suggest that the inhibitory effect triggered by high concentrations of amino acids was ameliorated in lines with reduced expression of *OsAAP3*. Enhanced translocation of Lys, Arg, His, Ala, Gln and Gly to the basal parts of the axillary buds in OE lines might contribute to the inhibition of growth of axillary buds. Thus, an appropriate level of expression of the transporter gene *OsAAP3* is required to support plant growth.

It has been reported that Lys can inhibit mitotic activity in the root apical meristem, and that exogenous Lys can reduce the length of the main root of *Arabidopsis* (Yang *et al*., 2014). Lys and Arg can be transported by the amino acid transporter AtAAP3 in *Arabidopsis*. The root system of *AtAAP3* mutant is highly developed and shows a larger number of long main roots and a higher density of lateral root (Marella *et al*., 2013). *OsGS1.2*, which is a major gene for nitrogen assimilation, has been shown to promote axillary bud outgrowth and increase tiller numbers in rice (Obara *et al*., 2004; Ohashi *et al*., 2017). In contrast, bud outgrowth marker gene *OsFC1* is required for axillary buds outgrowth and has been reported to inhibit rice tiller number (Minakuchi *et al*., 2010). In our study, expression of *OsGS1.2* was down‐regulated in OE lines and up‐regulated in Ri lines (Figure 3d); however, expression of *OsFC1* was up‐regulated in OE lines and down‐regulated in Ri lines (Figure 3e). Furthermore, it was reported that down‐regulation of *OsCKX2* expression increases tiller number and improves rice yield in rice (Yeh *et al*., 2015). Our results showed that higher expression of *OsCKXs* in OE lines and lower expression of *OsCKXs* in Ri lines than that in ZH11 (Figure S7) indicated that altered expression of *OsAAP3* controlled axillary bud outgrowth possibly by regulating CK pathway in the axillary bud.

Additionally, amino acids are used for basic metabolism and protein synthesis in the leaf, and then modulate plant growth (Yadav *et al*., 2015). Our results showed that total free amino acid concentrations in the leaves of OE lines were decreased significantly, but increased significantly in the leaves of Ri lines (Figure 4a). Starting with less outgrowth buds, less tiller numbers and poor plant growth of OE lines (Figure 2a) might result from lacking of amino acids. We further indicated that repression of *OsAAP3* could promote bud outgrowth and seedling growth, and trigger the development of a larger number of tillers through regulating the allocation and utilization of amino acids by enhancing the ability of nitrogen balance.

### Inhibition of *OsAAP3* expression increases grain yield and NUE in *Japonica* rice varieties

Haplotype‐level association analysis is an important tool for molecular plant breeding (Han *et al*., 2016). It has been reported that the *NRT1.1B‐indica* allele can increase tiller numbers per plant, enhance grain yield per plant and increase yield per plot under high N level (Hu *et al*., 2015). In the present study, we analysed the promoter and coding regions of *OsAAP3* in a diverse collection of 524 *O. sativa* landraces. Some differences were found in SNPs in the promoter sequences of *OsAAP3* of *Japonica* (Hap1 and Hap2) and *Indica* (Hap4) accessions. Expression of *OsAAP3* was higher in the *Japonica* accessions. *Japonica* rice has well‐known nutritional qualities but produces fewer tillers and has a lower NUE than *Indica* rice (Hu *et al*., 2015; Koutroubas and Ntanos, 2003). Reduced expression of *OsAAP3* can increase yield and NUE and does not affect nutritional quality. Therefore, inhibiting expression of *OsAAP3* in *Japonica* rice may be important for improving tillering and grain yield. CRISPR technology is an effective method for inhibiting gene expression in plants (Ma *et al*., 2015; Yin *et al*., 2017). Our results indicated that tiller numbers and grain yield (Figure 7) increased significantly in *OsAAP3*‐CRISPR lines on a *Japonica* ZH11 or KY131 background. Thus, inhibition of *OsAAP3* expression may contribute to an improved grain yield and NUE for *Japonica* varieties.

## Experimental procedures

### Construction of *OsAAP3* altered expression and promoter‐GUS vector

To construct *OsAAP3* overexpressing plants, a 1464 bp *OsAAP3* cDNA containing the open reading frame (ORF) of *OsAAP3* (LOC_Os06g36180) was inserted downstream of the *35S* promoter in pCAM1306 using *Kpn* I and *Xba* I to produce the plasmid *p35S*‐*OsAAP3*. To generate the *OsAAP3*‐RNAi construct, two fragments of *OsAAP3* cDNA (309 bp) were amplified by PCR using the primers listed in Table S1 and transferred downstream of the *Ubi‐1* promoter in the rice RNAi vector pTCK303 (Wang *et al*., 2004) using *BamH* I/*Kpn* I, and *Spe* I/*Sac* I, respectively, to generate the *OsAAP3*‐RNAi vector *pOsAAP3i*. To analyse the *OsAAP3* promoter, a 2179‐bp *OsAAP3* promoter fragment was generated by PCR using the primers shown in Table S1, and inserted in front of the β‐glucuronidase (GUS) coding region in pCAMBIA1391Z with *Hind* III and *Nco* I to generate the *pOsAAP3*‐*GUS* plasmid.

### Construction of *OsAAP3* CRISPR vector

The *OsAAP3* CRISPR vector construct was prepared using CRISPR/Cas9‐based multiplex genome editing for monocot and dicot plants (Ma *et al*., 2015). Two target sequences of *OsAAP3* were designed and added to U6IPAT/U6OPST or U3IPAT/U3OPST primers. A 422‐bp fragment from target sequence 1 was amplified by PCR using U6OPST and AAP3‐U6IPAT_1_ primers and the U6 plasmid. In the same way, a 348‐bp fragment was amplified by PCR from target sequence 2 using ZRO589 and AAP3‐U3IPAT_2_ primers with the U3 plasmid. A 476‐bp fragment from target sequence 1 was amplified by PCR using AAP3‐U6OPST and U6IPAT primers with the U6 plasmid. Similarly, a 463‐bp fragment from target sequence 2 was amplified by PCR using AAP3‐U3IPST and ZRO282 primers with the U3 plasmid. Complete U6 and U3 fragments were amplified by fusion PCR containing U6 promoter‐sgRNA and U3 promoter‐sgRNA. The U6 fragment was digested with *Kpn* I, and the U3 fragment was digested with *Kpn* I and *Sac* I and then the U6 and U3 fragment were connected. The complete U6 and U3 sequences of 1600 bp were amplified by PCR using U6OPST and ZRO282 primers with the U6–U3 plasmid. Finally, the complete U6–U3 fragment of 1600 bp was inserted into the Per8‐Cas9 vector using *Kpn* I and *Sac* I and cloning kits.

### Acquisition and detection of transgenic plants

All of the above‐mentioned constructs were introduced into *Agrobacterium tumefaciens* strain *EHA*105 (Hiei *et al*., 1997). The *Japonica* rice variety Zhonghua 11 (ZH11) and Kongyu 131 (KY131) were transformed by *Agrobacterium*‐mediated transformation with 50 mg/L hygromycin for selecting transgenic calli (Hiei *et al*., 1997). The T_2_ homologous transgenic lines (overexpression and RNAi) with altering expression were selected using PCR and the primers listed in Table S1. The T_0_ CRISPR transgenic lines were selected using PCR sequencing and the corresponding primers in Table S1.

### Hydroponic culture and field experiments

Hydroponic experiments were conducted using basic rice culture solution (Yoshida *et al*., 1976) without N under natural rice growth conditions; the N content was adjusted in each experiment. To analyse the phenotype of *OsAAP3* transgenic plants in the presence of different concentrations of NH_4_NO_3_, seedlings were cultivated in basic nutrient solution supplemented with 0.25 mm, 0.5 mm, 1.0 mm, 2.0 mm or 4.0 mm NH_4_NO_3_. To investigate the effect of each amino acid on the phenotype of *OsAAP3* transgenic plants, seedlings were grown in basic nutrient solution with 1.0 mm NH_4_NO_3_ as the N source for 1 week, then transferred to basic nutrient solution supplemented with 1.0 mm NH_4_NO_3_ and each amino acid as the N source. A collection of 524 *O. sativa* landraces (Chen *et al*., 2014) was used in this study. Tiller numbers were counted at the filling stage over three seasons from 2014 to 2017. Field experiments were carried out in an experimental field at Wuhan Institute of Bioengineering, China, during the rice growing season.

### GUS staining and quantitative RT‐PCR analysis

Histochemical GUS staining was performed according to Jefferson *et al*. (1987). The stained tissues were rinsed and fixed in FAA (formalin‐acetic acid‐70% ethanol [1:1:18]) at 4 °C for 24 h. Then the stained materials were observed using stereo microscope. Total RNA was extracted using TRIzol reagent according to the manufacturer's instructions (Invitrogen, http://www.invitrogen.com). First‐strand cDNA was synthesised from 3 μg of total RNA from each sample using M‐MLV reverse transcriptase (Promega, http://www.promega.com). Quantitative RT‐PCR (qRT‐PCR) was performed to monitor gene expression, and *OsActin1* (LOC_Os03g50885) was used as a control. qRT‐PCR was carried out in the presence of the double‐strand DNA‐specific dye SYBR Green (Takara, China) and monitored in real time with a 7500 qRT‐PCR system (Applied Biosystems, Singapore).

### Nitrogen and amino acid analysis

Total free amino acid concentrations were measured by the ninhydrin method (Fang *et al*., 2013). Single free amino acid concentrations were measured using HPLC with an amino acid analyser L‐8800 HITACHI. The samples were prepared as follows: 1.0 g rice tissue was placed in 80% ethanol (10.0 mL) at 80 °C in a water bath for 20 min; this step was repeated twice. The collected extracts were placed at 80 °C in a drying oven to remove the ethanol, and 1 mL 0.5 m NaOH was used to dissolve the sediment. The solution was centrifuged at 12000 ***g*** for 15 min. The supernatant was collected and filtered through a filter membrane (2 μm); 0.8 mL of each filtrate was analysed using the amino acid analyser. Total nitrogen content and total protein content were determined using the semimicro Kjeldahl method using a nitrogen analyser (Smart Chem 200, Westco, Italy). Nitrogen utilization efficiency was determined from the formula:

$$\begin{array}{cc} \text{NUE}(\%) & =[\text{grain yield (g)/(grain nitrogen content (g)}\\ & \;+\text{straw nitrogen content (g)}]\times 100. \end{array}$$

### Bud outgrowth analysis

Germinated seeds were transferred into a hydroponic culture box with sterile water and cultured at 28 °C and 60% relative humidity under white light with a 16‐h light/8‐h dark photoperiod for 3 day. They were then grown with rice culture solution for an additional 6 d under the same conditions. Then, seedlings for bud outgrowth analysis were placed in rice culture solution in a glasshouse at 32 °C under a high voltage sodium lamp (400 w) for 14 h, and at 25 °C in the dark for 10 h in the evening. The nutrient solution was renewed every 3 day. The length of bud outgrowth was measured using stereo microscope with ImageJ software.

### SNP database

In total, 524 accessions were genotyped via sequencing (Chen *et al*., 2014). SNP information is available on RiceVarMap (http://ricevarmap.ncpgr.cn/), which is a comprehensive database of rice genomic variations. SNP physical locations were obtained from the TIGR Rice Loci 6 genome in RiceVarMap. Haplotypes with an allele frequency of 0.01 or higher (five accessions) were used for association analysis via ANOVA. Duncan's multiple range test was applied to identify differences in SNP content between all possible haplotype pairs.

### Statistical analysis

For all treatments, the statistical differences are indicated by asterisks, and the Student's *t*‐test allowing the determination of the significance between two sets of data (transgenic line vs wild‐type ZH11 or KY131) is performed using the SPSS 10 software (IBM, Inc.). Significant levels: ***P <* *0.001; **P <* *0.01; * *P *<* *0.05.

## Supporting information


**Figure S1** Effect of different NH_4_NO_3_ concentrations on growth of the rice seedlings with altered expression of *OsAAP3*.
**Figure S2** The expression of *OsAAP3* is regulated by both amino acids Lys and Arg.
**Figure S3** Effect of different amino acids (Asp, Ser, Gly and Tyr) concentrations on outgrowth bud elongation of the rice seedlings with ZH11 and altered expression of *OsAAP3*.
**Figure S4** Effect of different amino acids (Thr, Ala, Val, Leu and Gln) concentrations on outgrowth bud elongation of the rice seedlings with ZH11 and altered expression of *OsAAP3*.
**Figure S5** Effect of different amino acids (Ile, Phe and His) concentrations on outgrowth bud elongation of the rice seedlings with ZH11 and altered expression of *OsAAP3*.
**Figure S6** Effect of different amino acids Lys and Arg on growth of the rice seedlings with altered expression of *OsAAP3*.
**Figure S7** The expression of *OsCKXs* in basal part of the rice seedlings with altered expression of *OsAAP3* grown for 3 weeks in basic nutrient solution with 1.0 mm NH_4_NO_3_ as the N source.
**Figure S8** The expression of *OsAAP3* in root and basal part of *Japonica* ZH11 and KY131 seedlings grown for 3 weeks in basic nutrient solution with 1.0 mm NH_4_NO_3_ as the N source.
**Table S1** List of the primers in this study.
