# Supplementary material for: Blocking amino acid transporter OsAAP3 improves grain yield by promoting outgrowth buds and increasing tiller number in rice
Source: Plant Biotechnol J. 2018 Mar 25;16(10):1710–22. doi: 10.1111/pbi.12907 (PMC6131477; doi:10.1111/pbi.12907)
Supplement: Supplementary file 1 — Figure S1 Effect of different NH4NO3 concentrations on growth of the rice seedlings with altered expression of OsAAP3. Figure S2 The expression of OsAAP3 is regulated by both amino acids Lys and Arg. Figure S3 Effect of different amino acids (Asp, Ser, Gly and Tyr) concentrations on outgrowth bud elongation of the rice seedlings with ZH11 and altered expression of OsAAP3. Figure S4 Effect of different amino acids (Thr, Ala, Val, Leu and Gln) concentrations on outgrowth bud elongation of the rice seedlings with ZH11 and altered expression of OsAAP3. Figure S5 Effect of different amino acids (Ile, Phe and His) concentrations on outgrowth bud elongation of the rice seedlings with ZH11 and altered expression of OsAAP3. Figure S6 Effect of different amino acids Lys and Arg on growth of the rice seedlings with altered expression of OsAAP3. Figure S7 The expression of OsCKXs in basal part of the rice seedlings with altered expression of OsAAP3 grown for 3 weeks in basic nutrient solution with 1.0 mm NH4NO3 as the N source. Figure S8 The expression of OsAAP3 in root and basal part of Japonica ZH11 and KY131 seedlings grown for 3 weeks in basic nutrient solution with 1.0 mm NH4NO3 as the N source. Table S1 List of the primers in this study. [file PBI-16-1710-s001.docx]

**Supporting information**


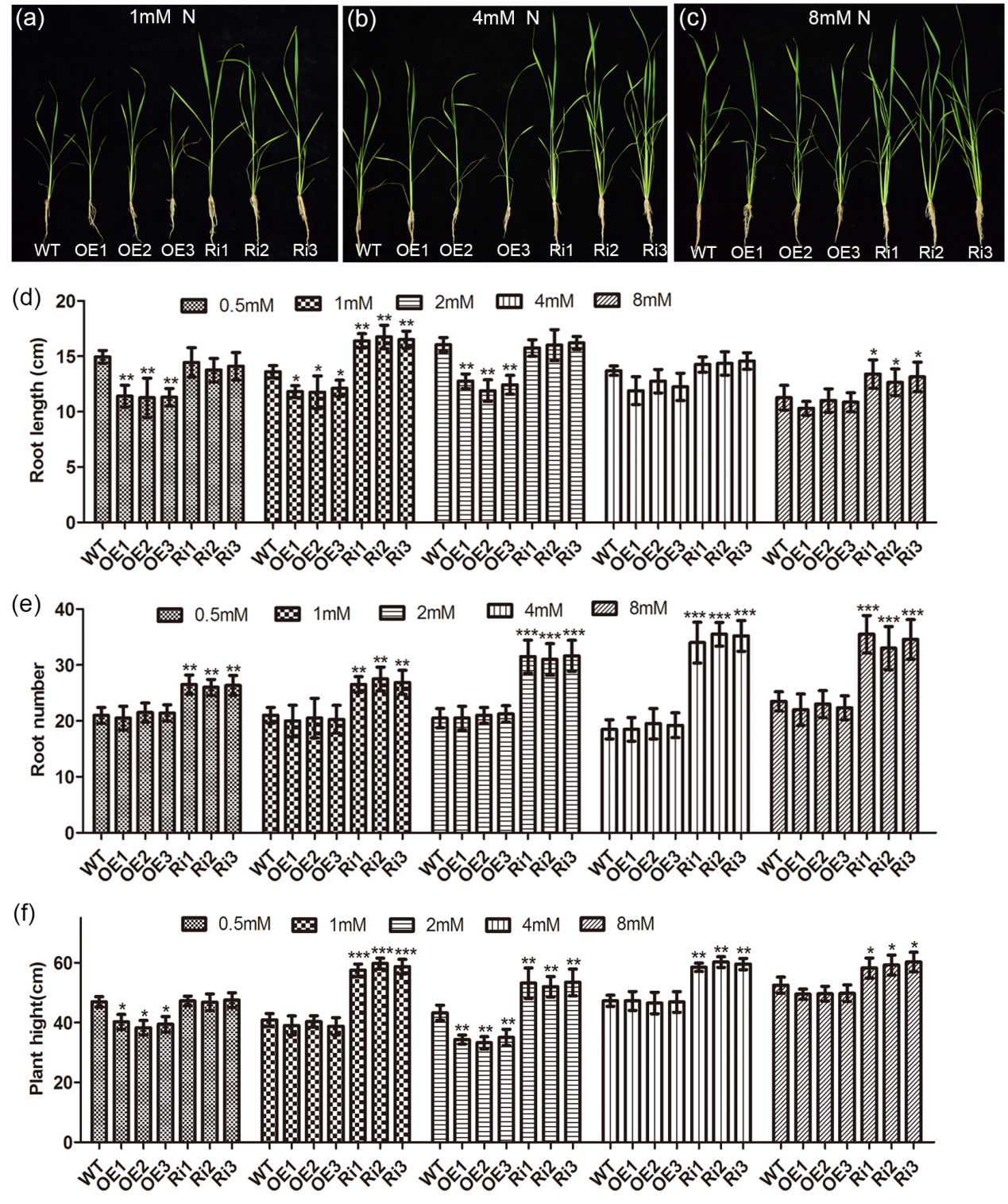


**Figure S1.** Effect of different NH_4_NO_3_ concentrations on growth of the rice seedlings with altered expression of *OsAAP3*. Seedlings of wild-type (WT, ZH11), *OsAAP3* overexpressing lines (OE1-OE3), *OsAAP3*-RNAi lines (Ri1-Ri3) were grown in nutrient solution containing different nitrogen concentration (0.5 mM, 1.0 mM, 2.0 mM, 4.0 mM, 8.0 mM). The phenotypes of seedlings were taken under 0.5 mM NH_4_NO_3_ (a), or 2.0 mM NH_4_NO_3_ (b), or 4.0 mM NH_4_NO_3_ (c) when the rice plants were grown for 28 days. The root length (d), root number (e), and plant height (f) were taken when the rice plants were grown for 28 days. The proportion of (a)-(c) is the same size. Values are means ± s.d. (n>10). Significant levels: ***P < 0.001; **P < 0.01; * P < 0.05.


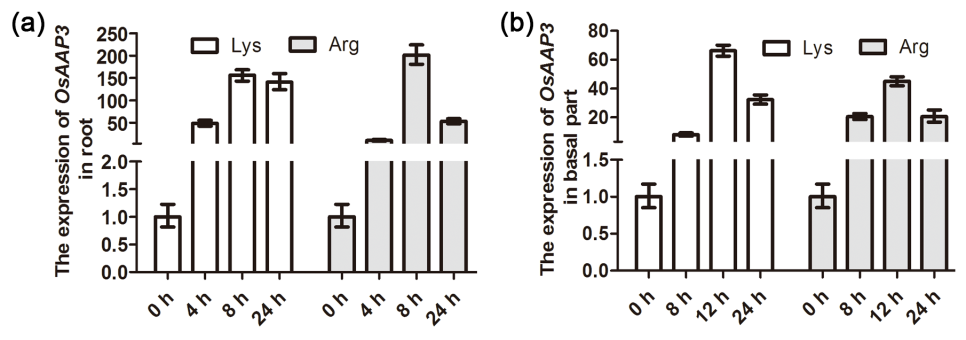


**Figure S2.** The expression of *OsAAP3* is regulated by both amino acid Lys and Arg. The expression of *OsAAP3* in the root (a) or in the basal part (b) with amino acid Lys or Arg treatments was measured. ZH11 seedlings were grown for three weeks in basic nutrient solution with 1.0 mM NH_4_NO_3_ as the N source then transferred to N-free basic nutrient solution for three days (N starvation). The N-starved seedlings were transferred to basic nutrient solution supplemented with 1.0 mM NH_4_NO_3_+0.5 mM Lys or 1.0 mM NH_4_NO_3_+0.5 mM Arg as the N sources. Values are means ± s.d. (n>3).


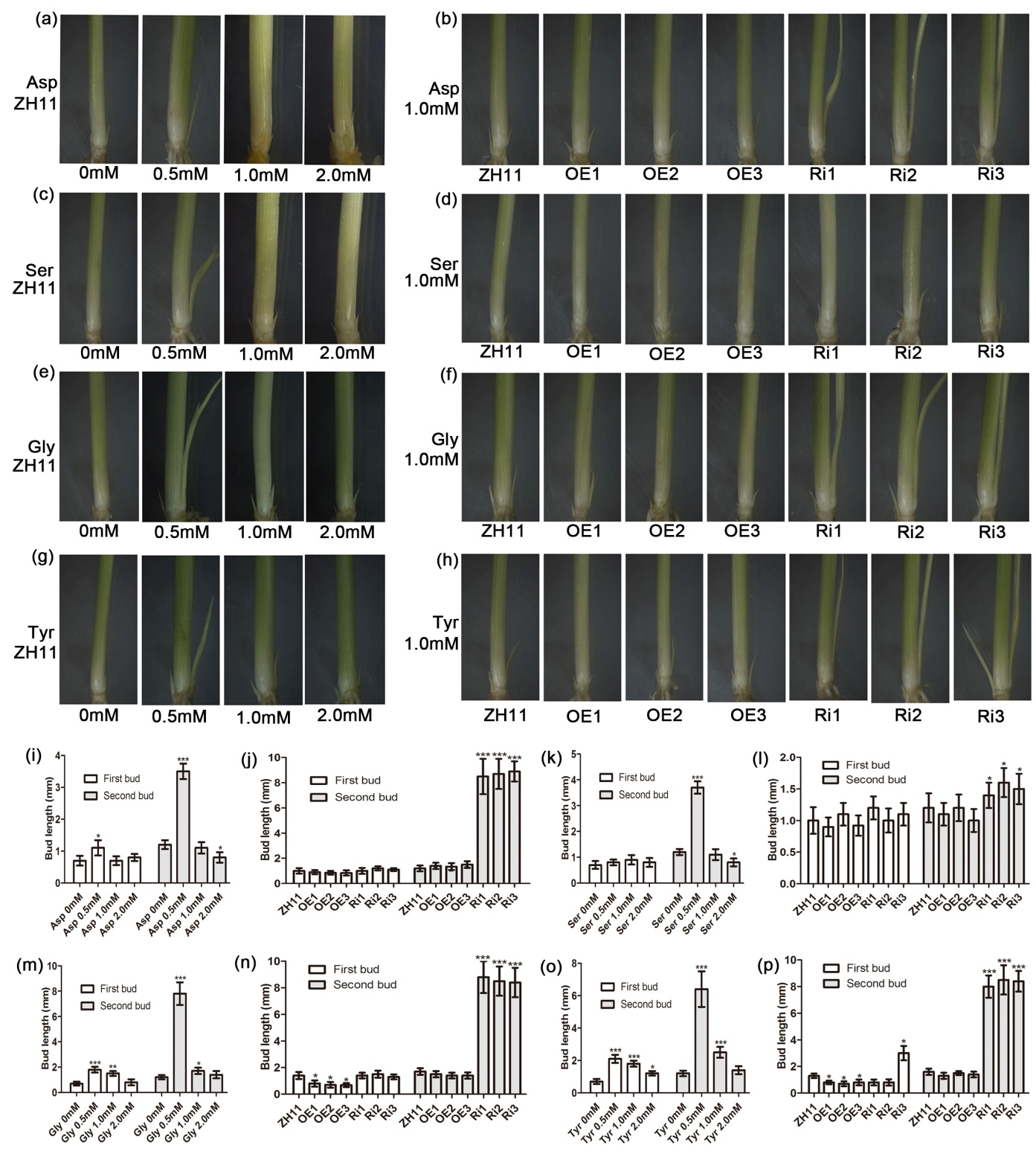


**Figure S3.** Effect of different amino acids (Asp, Ser, Gly and Tyr) concentrations on outgrowth bud elongation of the rice seedlings with ZH11 and altered expression of *OsAAP3*. Phenotypes (a-h) and bud length (i-p) analyses of outgrowth bud with 1.0 mM NH_4_NO_3_+amino acid concentration grown in hydroponic culture for 28 d. Phenotypes (a, c, e, g) and bud length (i, k, m, o) of outgrowth bud in ZH11 under 1.0 mM NH_4_NO_3_ containing 0-2.0 mM amino acids concentration (Asp, or Ser, or Gly, or Tyr). Phenotypes (b, d, f, h) and bud length (j, l, n, p) of outgrowth bud in ZH11, OE1-OE3 lines, and Ri1-Ri3 lines under 1.0 mM NH_4_NO_3_ containing 1.0 mM amino acid Asp, or Ser, or Gly, or Tyr. The proportion of (a)-(h) is the same size. Values are means ± s.d. (n>10). Significant levels: ***P < 0.001; **P < 0.01; * P < 0.05.


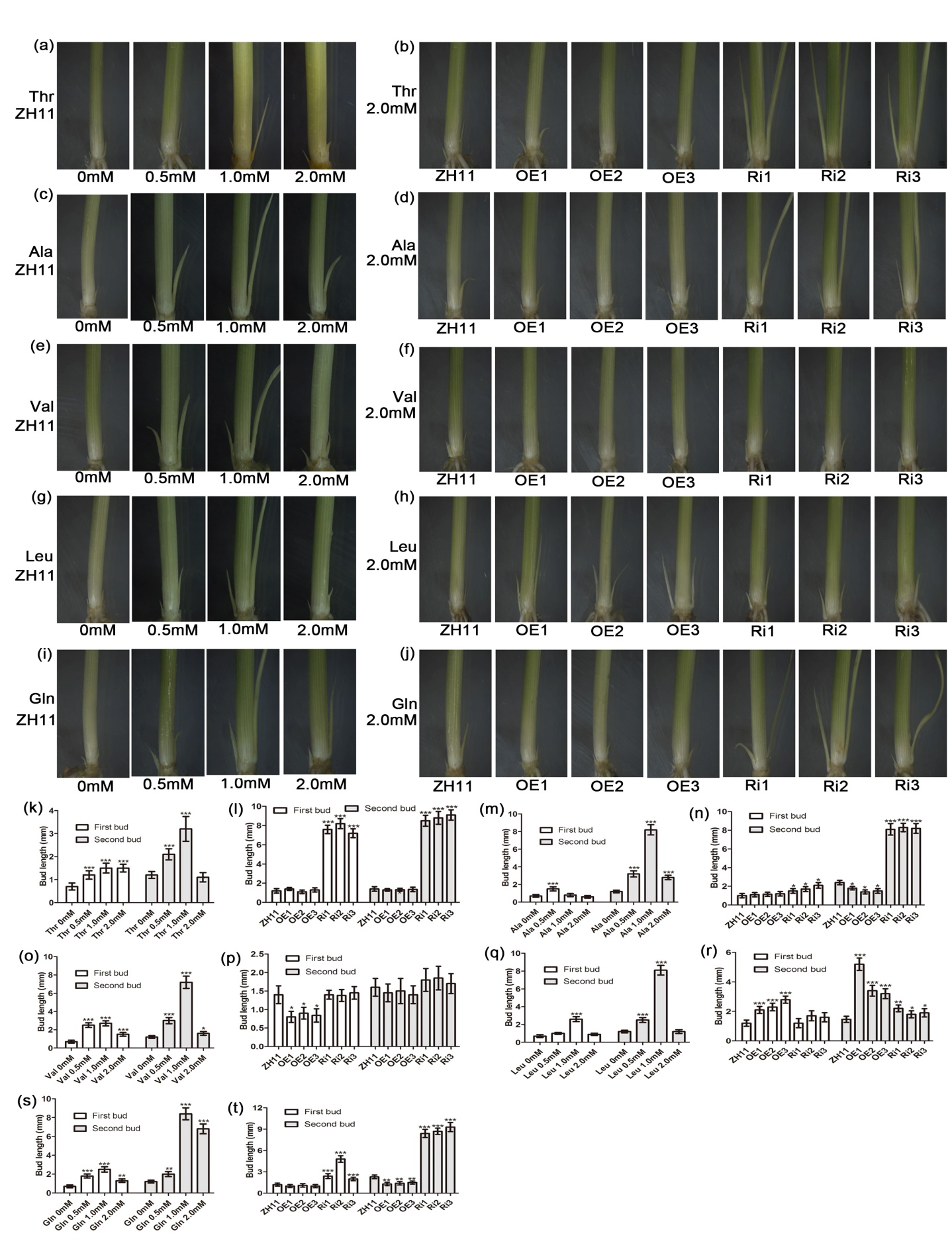


**Figure S4.** Effect of different amino acids (Thr, Ala, Val, Leu and Gln) concentrations on outgrowth bud elongation of the rice seedlings with ZH11 and altered expression of *OsAAP3*. Phenotypes (a-j) and bud length (k-t) analyses of outgrowth bud with 1.0 mM NH_4_NO_3_+amino acid concentration grown in hydroponic culture for 28 d. Phenotypes (a, c, e, g, i) and bud length (k, m, o, q, s) of outgrowth bud in ZH11 under 1.0 mM NH_4_NO_3_ containing 0-2.0 mM amino acids concentration (Thr, or Ala, or Val, or Leu, or Gln). Phenotypes (b, d, f, h, j) and bud length (l, n, p, r, t) of outgrowth bud in ZH11, OE1-OE3 lines, and Ri1-Ri3 lines under 1.0 mM NH_4_NO_3_ containing 2.0 mM amino acid Thr, or Ala, or Val, or Leu, or Gln. The proportion of (a)-(j) is the same size. Values are means ± s.d. (n>10). Significant levels: ***P < 0.001; **P < 0.01; * P < 0.05.

**
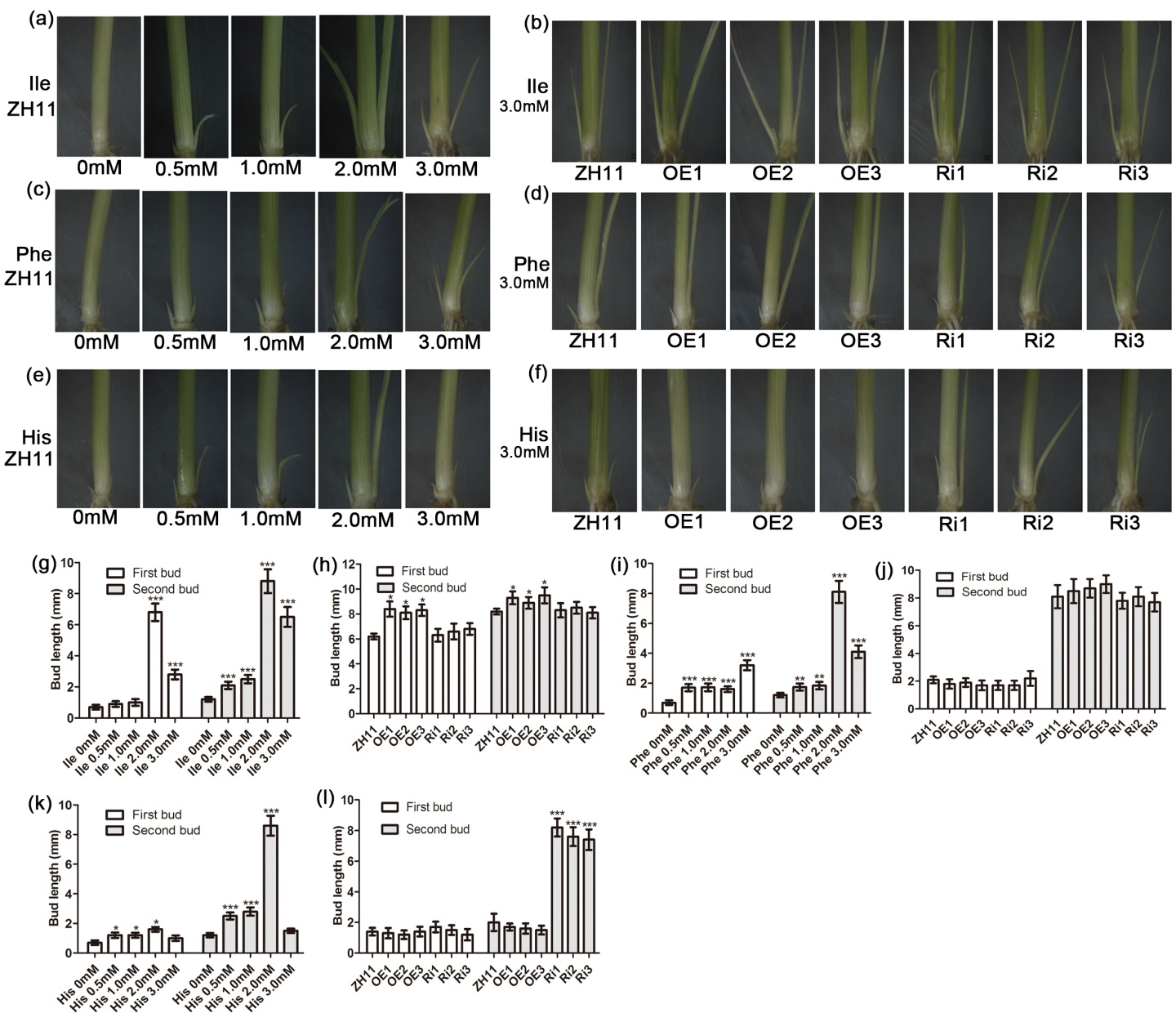
**

**Figure S5.** Effect of different amino acids (Ile, Phe and His) concentrations on outgrowth bud elongation of the rice seedlings with ZH11 and altered expression of *OsAAP3*. Phenotypes (a-f) and bud length (g-l) analyses of outgrowth bud with 1 mM NH_4_NO_3_+amino acid concentration grown in hydroponic culture for 28 d. Phenotypes (a, c, e) and bud length (g, i, k) of outgrowth bud in ZH11 under 1.0 mM NH_4_NO_3_ containing 0-3.0 mM amino acids concentration (Ile, or Phe, or His). Phenotypes (b, d, f) and bud length (h, j, l) of outgrowth bud in ZH11, OE1-OE3 lines, and Ri1-Ri3 lines under 1.0 mM NH_4_NO_3_ containing 3.0 mM amino acid Ile, or Phe, or His. The proportion of (a)-(f) is the same size. Values are means ± s.d. (n>10). Significant levels: ***P < 0.001; **P < 0.01; * P < 0.05.


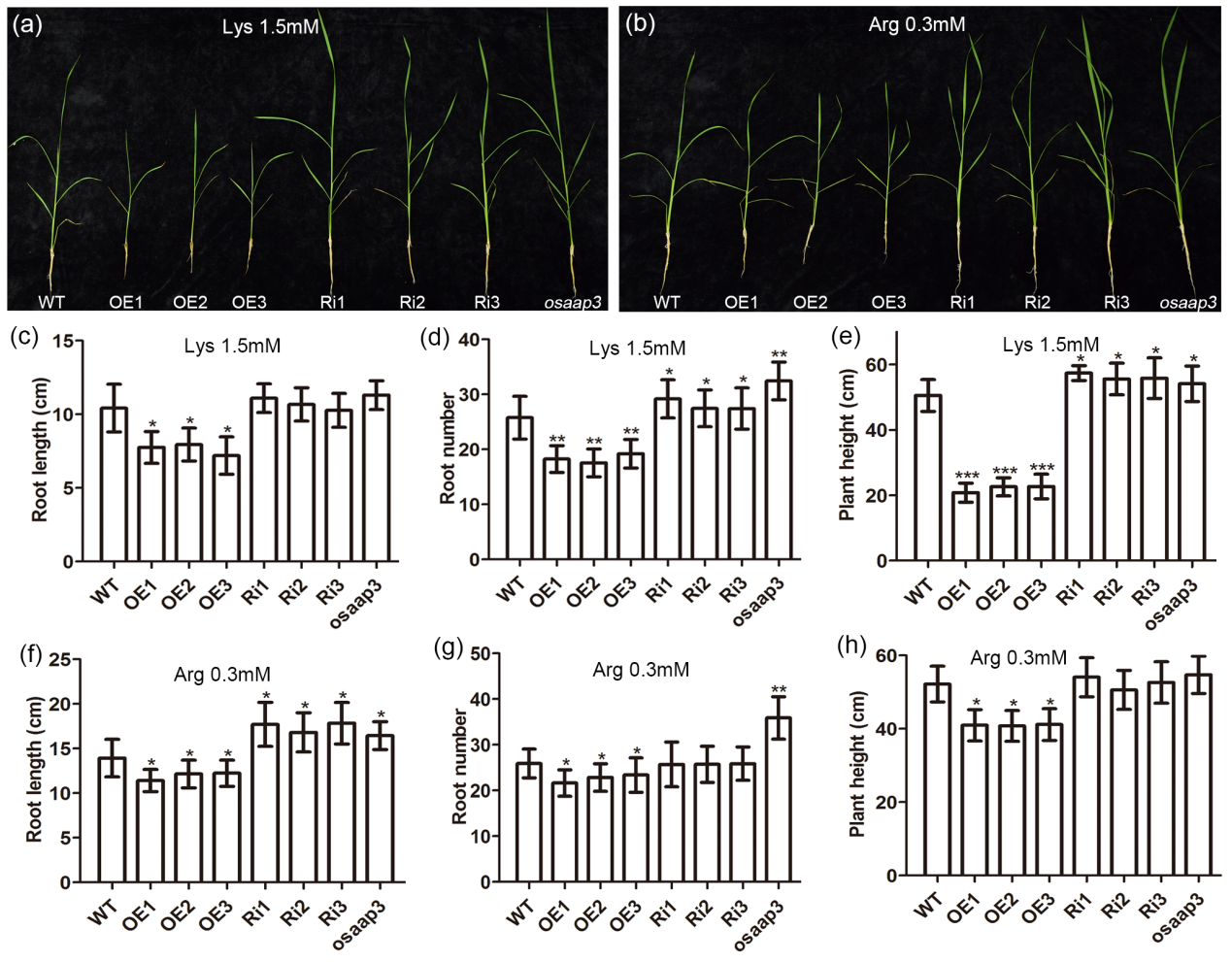


**Figure S6.** Effect of different amino acid Lys and Arg on growth of the rice seedlings with altered expression of *OsAAP3*. Seedlings of wild-type (WT, ZH11), *OsAAP3* overexpressing lines (OE1-OE3), *OsAAP3*-RNAi lines (Ri1-Ri3) were grown in nutrient solution containing 1.0 mM NH_4_NO_3_ and different amino acid concentration. The phenotypes of seedlings were taken under 1 mM NH_4_NO_3_ and 1.5 mM Lys (a), or 1.0 mM NH_4_NO_3_ and 0.3 mM Arg (b) when the rice plants were grown for 28 days. The root length (c), root number (d), and plant height (e) were taken 1.0 mM NH_4_NO_3_ and 1.5 mM Lys when the rice plants were grown for 28 days. The root length (f), root number (g), and plant height (h) were taken 1.0 mM NH_4_NO_3_ and 0.3 mM Arg when the rice plants were grown for 28 days. The proportion of (a)-(b) is the same size. Values are means ± s.d. (n>10). Significant levels: ***P < 0.001; **P < 0.01; * P < 0.05.


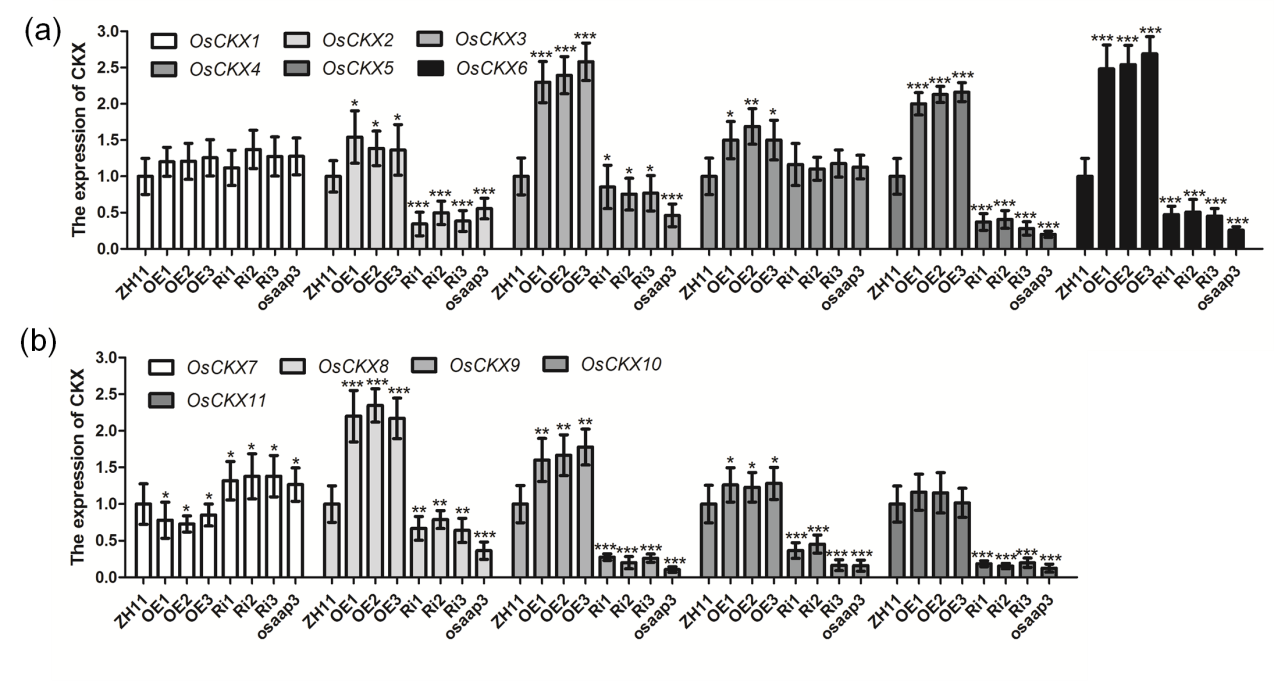


**Figure S7.** The expression of *OsCKXs* in basal part of wild-type ZH11, *OsAAP3* overexpressing lines (OE1-OE3), *OsAAP3*-RNAi lines (Ri1-Ri3), *osaap3* mutant seedlings grown for three weeks in basic nutrient solution with 1.0 mM NH_4_NO_3_ as the N source. Values are means ± s.d. (n>3). Significant levels: ***P < 0.001; **P < 0.01; * P < 0.05.


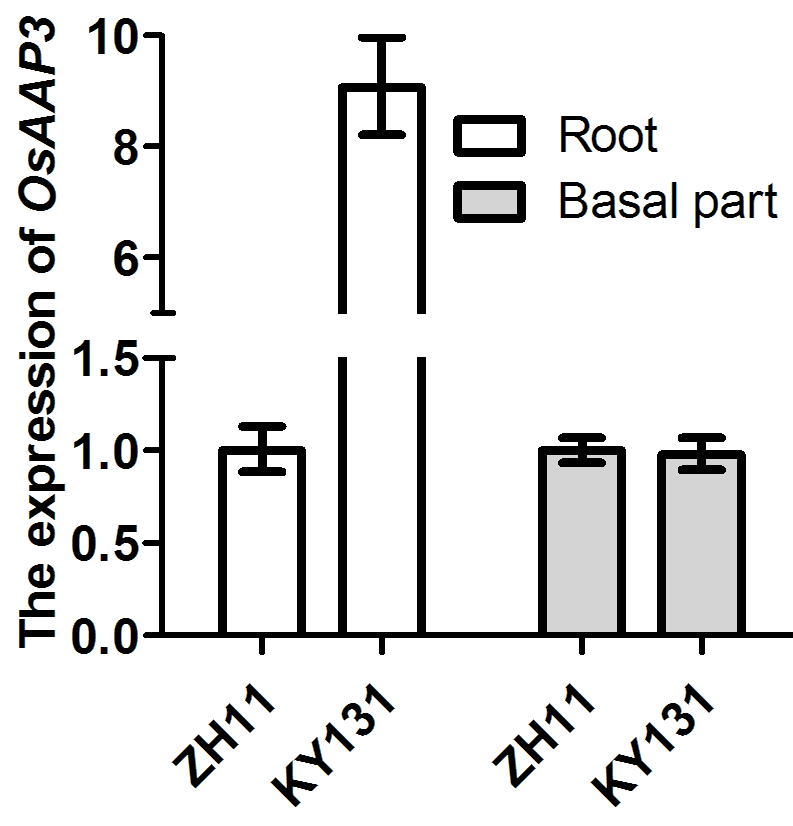


**Figure S8.** The expression of *OsAAP3* in root and basal part of *Japonica* ZH11 and KY131 seedlings grown for three weeks in basic nutrient solution with 1.0 mM NH_4_NO_3_ as the N source. Values are means ± s.d. (n>3).

**Table S1.** List of the primers in this study.

| **Names** | **Forward sequence (5' - 3')** | **Reverse sequence (5' - 3')** |
| --- | --- | --- |
| OsAAP3-OE | AACCATGGGGTACCATGGCGAAGGACGTGGAGATGGCG | AAACTAGTTCTAGACGACTTGGTCTTGAAGGGGACGTA |
| OsAAP3-Ri-1 | ACTAGTATGGCGAAGGACGTGGAGATGGCG | GAGCTCATCGCCAGTGCGGTAGCAGTC |
| OsAAP3-Ri-2 | GGTACCATGGCGAAGGACGTGGAGATGGCG | GGATCCATCGCCAGTGCGGTAGCAGTC |
| pAAP3:GUS | TTAAGCTTGTTCCTATCAAGAGCTCCAAGG | TTGGATCCTCGAAGCGATTTGAATGGACG |
| OsAAP3-U6IPST1 | GCGGGCTCGGAGTTCATCACCGTTTTAGAGCTAGAAATAGCAAGTTA | GGTGATGAACTCCGAGCCCGCAACCTGAGCCTCAGCGCAGC |
| OsAAP3-U3IPST2 | ATACAGCGGTACACGTCCAGGGTTTTAGAGCTAGAAATAGCAAGTTA | CCTGGACGTGTACCGCTGTATGCCACGGATCATCTGCACAACTC |
| qOsActin | CGGTGTCATGGTCGGAAT | GCTCGTTGTAGAAGGTGT |
| qOsAAP3 | GCGGAGAACAAGACGATGAA | ATGGGCTGGCAGAACACC |
| qOsGS1.2 | TGTTTCTCCTCATCCCTGC | TCACAGTCCTCGCTTTGC |
| qFC1 | TCGTCCACCAATCTTGTGAGCACC | GTTGGCGAACGCCATGATCACGTC |
| qOsCKX1 | TCAACAAATCCAAGTGGGATGCGG | TCGCAGAACCTCAGTATCCTCCTGT |
| qOsCKX2 | GTCAGTGGAGGGGCGGTA | CGTTGGAAATCTGGGGGC |
| qOsCKX3 | ATGAGCAATCCCTTCACAGCTCCT | TGACTTCCACGACCTGTTCCACAT |
| qOsCKX4 | GACCGACTACCTCCATCTCACA | GGTTGACATTGCTGACCTGC |
| qOsCKX5 | AGGGCCTAATCAACAACTGGAGGT | GGTGGAGTCGTCGTAATTCTTGGT |
| qOsCKX6 | ACAAGTCCAGCTCAATCGGACACT | TCCCGCAAGCCTATCAATGTCGAA |
| qOsCKX8 | CATCAGTGGCCAAACGTTCAAGCA | AGTTGGAGTGGAATCCTTGCCCTT |
| qOsCKX9 | GCCTTCCTCCTTATTCCACA | TGGGACCATTGTTACTGTCTTTC |
| qOsCKX10 | ACTTGAACCAGCTCCGAAAAGA | TGCAGAGAAGAAGGAAGACGAC |
| qOsCKX11 | TGGCGAGATCTTCTACCTGGTG | CAATGATTGCGTTGTTCTGCGCCA |
